# Application of Light-Responsive Nanomaterials in Bone Tissue Engineering

**DOI:** 10.3390/pharmaceutics17010098

**Published:** 2025-01-13

**Authors:** Aiguo Liu, Chenxu Wang, Shuang Deng, Sitong Zhang, Ziwen Zhao, Han Xiao, Ting Ying, Chengqing Yi, Dejian Li

**Affiliations:** 1 Department of Orthopedics, The First Affiliated Hospital of Henan University, Kaifeng 475000, China; doctorlag@163.com (A.L.); wangchenxu255@163.com (C.W.); 2 Department of Orthopedics, Shanghai Pudong Hospital, Fudan University Pudong Medical Center, 2800 Gongwei Road, Pudong, Shanghai 201300, China; dengshuang1214@163.com (S.D.); zhangsitong100@163.com (S.Z.); dr_zhaoziwen@163.com (Z.Z.); xiaohan970816@163.com (H.X.); 18321906659@163.com (T.Y.)

**Keywords:** photoresponsive, nanometer material, bone regeneration, photothermal therapy, photodynamic therapy

## Abstract

The application of light-responsive nanomaterials (LRNs) in bone tissue engineering shows broad prospects, especially in promoting bone healing and regeneration. With a deeper understanding of the mechanisms of bone defects and healing disorders, LRNs are receiving increasing attention due to their non-invasive, controllable, and efficient properties. These materials can regulate cellular biological reactions and promote bone cell adhesion, proliferation, and differentiation by absorbing specific wavelengths of light and converting them into physical and chemical signals. In addition, the unique surface morphology and biocompatibility of LRNs enable them to effectively load drugs in bone tissue engineering, achieve precise release, and optimize the bone regeneration process. Through photothermal and photodynamic therapy, these materials also possess antibacterial properties and can play an important role in the repair of infectious bone defects. Although LRNs have shown significant advantages in bone tissue regeneration, a series of challenges still need to be overcome to achieve their widespread and effective clinical applications. This article summarizes the basic principles, classification, and potential applications of LRNs in bone tissue regeneration, aiming to provide reference for future research and clinical applications.

## 1. Introduction

Bone defects and impaired bone healing are frequently the result of inflammatory processes, traumatic injuries, and the formation of tumors [1]. These conditions have a considerable impact on the quality of life of patients. In specific physiological and pathological conditions, such as advanced age, diabetes, and osteoporosis, the speed and efficacy of osteogenesis may be diminished, consequently exacerbating the challenge of bone healing [2]. The promotion of early bone healing and the reduction of infection risk have emerged as key research areas. In recent years, the synthesis of stimuli-responsive nanomaterials (such as light-, magnetic field-, and ultrasound-responsive materials) has attracted considerable attention in the field of bone tissue regeneration, following the rapid development of nanobiomaterials [3]. Among these external stimuli, light stimulation is particularly advantageous due to its non-invasive nature, ease of remote manipulation, and highly precise spatiotemporal response to specific wavelengths, which enables precise regulation of cellular signaling systems [4].

The potential applications of light-responsive nanomaterials (LRNs) in bone tissue regeneration are promising. The classification of LRNs is based on their properties and characteristics, which can be divided into two categories: organic and inorganic. Both categories have been demonstrated to play a significant role in the promotion of bone tissue regeneration [5]. Organic LRNs typically demonstrate favorable biocompatibility and degradability, whereas inorganic LRNs typically exhibit superior mechanical properties and biological activity [6]. By conducting comprehensive research on the characteristics of these materials and their mechanisms of action in bone healing, it is anticipated that new strategies for clinical bone repair will be developed, the advancement of bone tissue engineering will be facilitated, and the efficacy and quality of life of patients will be enhanced.

**Figure 1:**
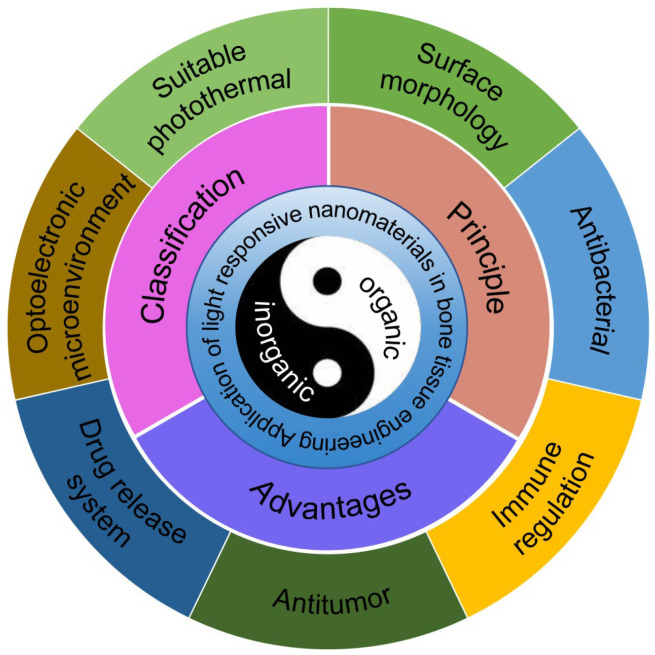
Application of light-responsive nanomaterials in bone tissue engineering.

This article provides a concise overview of LRNs and their categorization through a systematic review of the literature. It also presents an analysis of their potential applications in bone tissue regeneration, discusses the current challenges and future developments, and offers a reference point for the utilization of LRNs in clinical bone tissue regeneration (Figure 1).

## 2. Overview of LRNs

### 2.1. The Principle of LRNs

**Figure 2:**
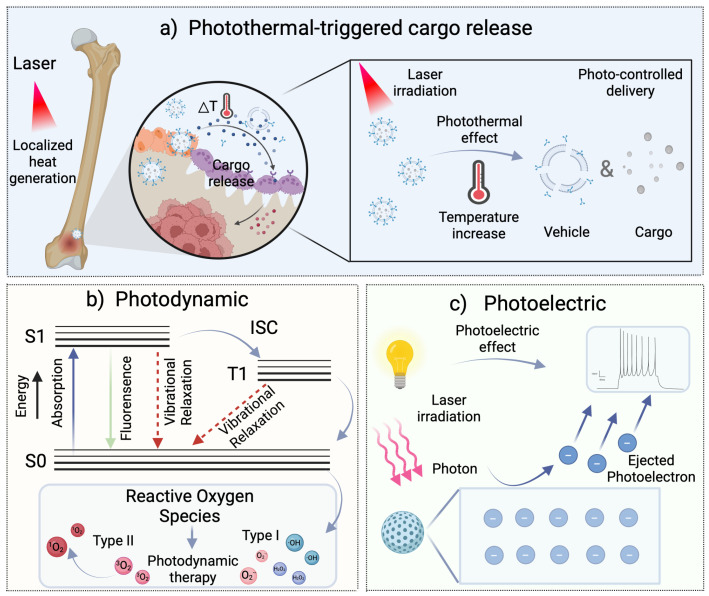
Photoresponsive nanomaterials utilized for bone tissue engineering: (**a**) photothermal effects of LRNs and photothermal-triggered release of bioactive factors. (**b**) Photodynamic effects of LRNs to generate ROS for biomedical applications. (**c**) Photoelectric effects of LRNs. Produced with Biorender.com.

The fundamental principle underlying the photoresponsive behavior of nanomaterials is their capacity to absorb and convert specific wavelengths of light. These materials are capable of converting light signals into physical and chemical signals when stimulated by specific light sources, resulting in alterations to their physicochemical properties and the generation of biological effects [7]. These nanomaterials comprise photosensitive components, including metal nanoparticles, dyes, or polymers, which undergo corresponding physical or chemical changes when exposed to specific wavelengths of light. To illustrate, LRNs can be incorporated with biomaterials or scaffolds to form photoreactive multifunctional scaffolds, exhibiting significant promise in bone tissue engineering, encompassing three primary mechanisms (Figure 2): (1) Photothermal Effect: Typically, photothermal agents are specific nanomaterials capable of efficiently absorbing light at certain wavelengths and converting it into thermal energy. This conversion causes a rapid local temperature rise around the photothermal agent. (2) Photodynamic Effect: Small-molecule photosensitizers absorb light energy, transitioning from the ground state to an excited state. This energy is transferred to surrounding oxygen molecules, generating reactive oxygen species (ROS) through redox reactions. (3) Photoelectric Effect: Under near-infrared light radiation, electrical signals are generated that depolarize cell membranes and stimulate intracellular calcium activity. Firstly, these scaffolds utilize photoreactive agents triggered by near-infrared light to perform localized photothermal therapy. Additionally, mild photothermal effects can also trigger the release of payloads within the scaffold to achieve therapeutic effects. Furthermore, the ROS produced by photodynamic therapy is lethal to bacteria or cancer cells. Finally, the photoelectric signals generated regulate osteogenic differentiation. These scaffolds not only generate ROS with antibacterial or antitumor effects but are also used for tissue regeneration, playing a crucial role in bone maturation, remodeling, and reconstruction [8]. The high spatiotemporal precision of LRNs allows for the regulation of biological responses at the cellular and tissue levels, thus facilitating the possibility of personalized therapy and minimizing adverse reactions to normal tissues [9]. A variety of light sources are available, including ultraviolet (10–400 nm), visible (400–700 nm), and NIR light (780–2526 nm). Each of these sources possesses distinctive characteristics. Of these, NIR light is particularly well suited to biomedical applications due to its minimal toxicity to normal tissues and higher tissue penetration depth [10]. In light of these characteristics, LRNs have demonstrated considerable potential for application in a range of fields, including tissue engineering, biological imaging, disease diagnosis, drug delivery, and targeted therapy [11,12,13].

### 2.2. Classification of LRNs

Nanomaterials can be classified into two main categories, organic and inorganic, based on their material properties [14,15]. The category of organic nanomaterials encompasses a range of carbon-based nanomaterials, including graphene and carbon nanotubes, as well as organic polymers, such as conjugated polymers, polypyrrole, and indocyanine green [16,17]. The superior biocompatibility of organic nanomaterials makes them a popular choice in the biomedical field. However, their relatively poor photostability limits their potential for long-term light exposure. Inorganic nanomaterials are further subdivided into several categories, including gold nanoparticles, phase transition nanoparticles, metal compounds (such as titanium (Ti) dioxide, molybdenum disulfide, tungsten diselenide, bismuth sulfide (BS), etc.), and single-element nanoparticles such as black phosphorus (BP) [18,19,20]. Inorganic nanomaterials typically demonstrate high photostability, conferring them with considerable advantages in optical applications. Nevertheless, these materials frequently necessitate modification to augment their biodegradability and facilitate a more expansive array of biomedical applications. Accordingly, in order to satisfy the diverse requirements of different applications, it is necessary for researchers to strike a balance between the characteristics of organic and inorganic nanomaterials, selecting the most appropriate material to meet the specific performance criteria.

### 2.3. The Advantages of LRNs in Bone Tissue Regeneration

The benefits of LRNs in bone tissue regeneration are primarily attributable to their distinctive physicochemical, biological, and photoresponsive characteristics. Firstly, as nanomaterials, LRNs exhibit high stability and good biocompatibility, which enables their prolonged existence in biological environments without eliciting adverse reactions [21]. In addition, the design flexibility and nanoscale size of these materials facilitate their binding with biomolecules, which in turn enhances their targeting and effectiveness in bone tissue engineering. Moreover, LRNs exhibit effective drug loading capabilities, facilitating precise drug delivery through diverse modalities, including direct injection, surface coating, and incorporation into bioactive scaffolds. This approach promotes bone tissue regeneration and repair [1].

Secondly, the photoresponsive properties endow these nanomaterials with unique functionalities. When illuminated by specific light sources, particularly NIR light, LRNs display remarkable photothermal and photodynamic effects, as well as the capacity for photo-controlled drug delivery [22]. These effects facilitate the precise regulation of bone tissue regeneration processes in a non-invasive manner. The ability to control the light source and regulate time and space allows for the implementation of personalized treatments, which consequently minimizes damage to surrounding tissues. It can therefore be concluded that LRNs have the potential to enhance the efficacy of drug release and bone regeneration whilst simultaneously reducing the risk of complications and improving the overall efficiency of the treatment process.

In conclusion, LRNs have demonstrated considerable potential for application in the field of bone tissue regeneration due to their distinctive physicochemical and biological properties, as well as their photoresponsive nature.

## 3. Application of LRNs in Bone Tissue Regeneration

### 3.1. Surface Morphology Promotes Bone Formation

LRNs, as a consequence of their distinctive nanosurface morphology, display a notable capacity to facilitate bone cell interactions in the context of bone tissue engineering. These materials have the capacity to enhance the adhesion, proliferation, and differentiation of bone cells by regulating their surface morphology and chemical properties [23]. The nanoscale concave–convex structure not only increases the surface area of the material but also provides a more favorable microenvironment, which in turn enhances the interaction between bone precursor cells and matrix [24]. The optimization of this microenvironment is of great importance for the growth and function of cells, as it has the potential to simulate the characteristics of the natural extracellular matrix, promoting the biological activity of cells [25].

**Figure 3:**
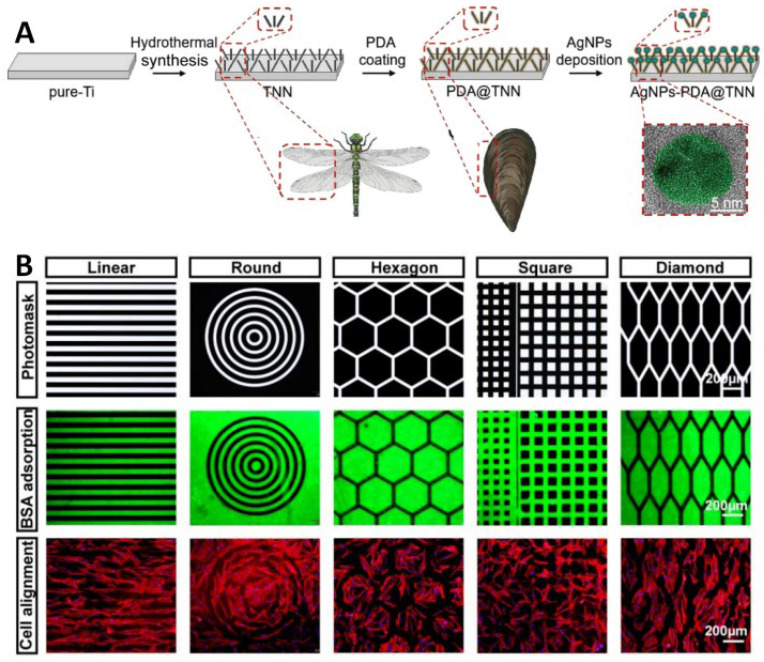
Surface morphology promotes bone formation: (**A**) AgNPs-PDA@TNN synthesis flowchart [26]. Reproduced content is open access. Copyright © 2024 The Authors. Published by Ivyspring International Publisher. (**B**) Micro pattern printing technology based on ultraviolet light on the surface of TiO_2_ nanorods: Fluorescent images of protein (fluorescein-conjugated BSA) patterns and fluorescent images of cell adhesion patterns after incubation for 12 h on the UV-patterned surfaces (hMSCs stained by rhodamine-phalloidin) [29]. Copyright © 2023 Wiley-VCH GmbH.

In their study, Chen et al. [26] employed a hydrothermal method to fabricate biomimetic insect wings with a disordered titanium dioxide nanoneedle (TNN) coating (Figure 3A). The surface of the TNN coating displays topographical characteristics analogous to nano-protrusions observed on insect wings, including those of cicadas and dragonflies [27]. This ingenious surface morphology has been shaped by natural evolution and selection, resulting in remarkable biological characteristics [28]. Polydopamine (PDA) and antibacterial silver nanoparticles (AgNPs) were coated on TNN, forming AgNPs-PDA@TNN composite materials. These composite materials have been observed to promote cell diffusion and enhance the osteogenic ability and matrix deposition of bone marrow stromal stem cells (BMSCs). The results of various imaging analyses indicate a significant increase in new bone formation within the body.

In a previous study, Lou et al. [29] fabricated micro patterns on the surface of Ti implants and tested the effect of TiO_2_ nanotube surface morphology on osteogenic properties in vitro and in vivo (Figure 3B). In vitro studies demonstrated that human mesenchymal stem cells (hMSCs) on the surface of linear patterns exhibited an elongated morphology and a parallel linear arrangement. Furthermore, their osteogenic differentiation ability was significantly enhanced under this surface morphology. The upregulation of key bone-specific functional genes in vitro can facilitate the formation of bone bonds between implants and host bones in vivo. Further mechanistic studies have demonstrated that linear patterned surfaces can facilitate cytoskeleton extension and, in turn, activate Yes Associated Protein (YAP)-related protein-mediated mechanical transduction pathways, which then promotes osteogenic differentiation of hMSCs.

Liu et al. [30] constructed a distinctive core–shell nanorod array in situ on a Ti substrate through a two-step hydrothermal process. Each strontium titanate (SrTiO_3_) nanorod was coated with a thin layer of nickel sulfide (NiS) on its surface, forming a composite structure comprising the NiS@SrTiO_3_ composite. The photoresponsive effect of the NiS layer, when combined with the physical perforation of SrTiO_3_ nanorods, exhibits a synergistic response when exposed to NIR irradiation. In vitro studies have demonstrated that the gradual exposure of SrTiO_3_ nanorods enhances surface hydrophilicity, facilitates cell adhesion, and stimulates the proliferation and differentiation of MC3T3-E1 cells. The in vivo evaluation demonstrated that the material exhibited superior osteogenic capacity, which facilitated the integration of Ti implants into bone tissue.

In a previous study, Belén et al. [31] proposed an adaptive electroforming method to obtain bio-shaped nanoporous Ti dioxide coatings on Ti6Al4V alloys. By precisely controlling the anodic polarization conditions, the structure demonstrates the formation of local nanopores arranged in a periodic assembly manner, which is beneficial for bone tissue-related treatments.

Zhao et al. [32] used tremilla-like zinc oxide (ZnO)@collagen type I (Col-I)-decorated Ti surfaces, thus forming ZnO@Col-I composite coatings. In vitro studies have demonstrated a notable elevation in alkaline phosphatase (ALP) activity and an enhancement in the expression levels of osteogenic genes, including runt-related transcription factor 2 (Runx2) and osteocalcin (OCN), in BMSCs. This suggests that the composite coating plays a pivotal role in facilitating the integration of implants within the bone. The long-term osteogenic data, analyzed by microcomputed tomography in the Sprague-Dawley rat femur model, demonstrates excellent osteogenic ability [32].

### 3.2. Antimicrobial-Enhanced Bone Regeneration

In the context of infectious bone defects, the ability of photoresponsive nanomaterials to exhibit photothermal and photodynamic therapeutic antimicrobial properties when exposed to specific light sources has attracted great interest.

Photothermal antimicrobial therapy is a process that uses photoresponsive nanomaterials to generate heat at specific wavelengths of light. This conversion of light energy into thermal energy serves to elevate local temperatures and disrupt the integrity of bacterial cell membranes. The application of elevated temperatures results in the denaturation of proteins within bacterial cells, leading to irreversible damage and the effective sterilization of the organism [33]. The ordered promotion of antibacterial and osteogenic light-responsive composite materials can achieve gradual functional regulation, meeting the needs of different stages of infectious bone defects.

Photodynamic antibacterial therapy is a process whereby LRNs undergo a photochemical reaction and redox process when illuminated, resulting in the production of reactive oxygen species (ROS) or other free radicals through energy transfer. This damages bacterial cell membranes, proteins, and DNA, ultimately leading to bacterial death. The method has the advantages of good controllability, low toxicity, and the absence of bacterial resistance [34,35].

**Figure 4:**
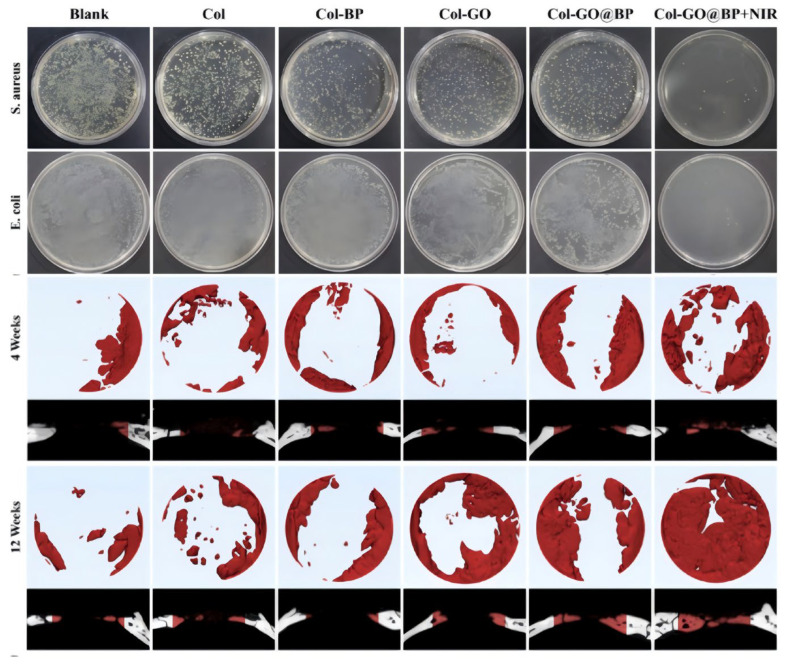
Evaluation of in vitro antibacterial activity and in vivo infectious bone defect repair: colony images on different scaffolds and representative coronal and sagittal micro CT reconstruction images at 4 and 12 weeks in different groups [34]. Copyright © 2024, American Chemical Society.

Chen et al. [36] integrated graphene oxide (GO) and BP nanoparticles into a reinforced porous collagen (Col) scaffold to prepare a multifunctional scaffold Col-GO@BP The bracket shows good photothermal effect of BP under NIR light irradiation. The photothermal effect accelerates the degradation of BP, enhances the reaction between PO_4_^3−^ and Ca^2+^, and promotes mineralization. The combined effect of the mild photothermal effect and sedimentary mineralization enhances BMSC osteogenic differentiation by upregulating heat shock proteins and activating the PI3K/Akt pathway. Furthermore, it has the capacity to destroy the bacterial envelope, exerting an antibacterial effect and markedly accelerating the process of infectious bone repair through its exceptional photothermal antibacterial performance and enhanced vascularization (Figure 4).

Liu et al. [30] synthesized a SrTiO_3_/NiS composite coating on the surface of Ti implants. This coating can be sterilized by the photothermal effect, undergoes gradual degradation, and releases nickel ions, which accelerate angiogenesis in bone defects. Furthermore, strontium carbonate nanorods have been demonstrated to promote the proliferation and differentiation of osteoblasts, which accelerates the osseointegration of Ti implants.

Wu et al. [37] coordinated BP nanosheets (BPs) with zinc sulfonate ligands (ZnL_2_), integrated ZnL_2_ BPs onto the surface of hydroxyapatite (HAP) scaffolds, and prepared the ZnL_2_-BPs@HAP. Upon exposure to NIR light irradiation, the photothermal effect generated by BP in the early stage increases the thermal sensitivity of bacteria surrounding the implant, reducing the risk of local infections. Subsequently, the application of photothermal temperatures and the release of biomineralized ions (Zn^2+^ and PO_4_^3−^), in accordance with the appropriate parameters, facilitate the process of osteogenesis in the subsequent stages of bone healing.

LIP@SrTiO_3_, designed by Liu et al. [38], is an L-arginine (LA)/new indocyanine green (NICG)-based anti-biofilm nanoplatform anchored to a SrTiO_3_ nanoarray on a Ti substrate by introducing PDA as an intermediate layer. NIR is used to excite NICG, generate ROS, react with LA to release nitric oxide molecules, and synergistically eliminate bacterial biofilms with the photothermal effect of PDA. The release of Sr^2+^ serves to promote osteoblast proliferation and differentiation, which facilitates the continuous optimization of the osteogenic microenvironment. In the rat bone defect model, the effective elimination of biofilms and reconstruction of the blood supply, in addition to the continuous optimization of the osteogenic microenvironment, significantly enhanced the osseointegration of the implant.

The composite material H-TNTs/f-Ti synthesized by Zhao et al. [39] using hydrogenated TiO_2_ nanotubes and Ti foil exhibits strong antibacterial and bone formation promoting abilities under light irradiation. On the one hand, the production of ROS through photocatalysis inhibits the proliferation of both Gram-negative and Gram-positive bacteria, creating a favorable environment for osteogenesis. On the other hand, the nanostructure of the material can accelerate the adhesion of initial proteins and promote the proliferation of MC3T3-E1 cells.

The antibacterial effects of photothermal and photodynamic antibacterial agents can reduce local infections, which reduces inflammatory reactions and creates a favorable microenvironment for bone defect repair. Nevertheless, in order to achieve optimal antibacterial effects, photothermal antibacterial agents typically necessitate higher temperatures, whereas photodynamic antibacterial agents require the generation of a substantial quantity of ROS, which may potentially result in damage to surrounding normal tissues [40]. The current research combines photothermal and photodynamic antibacterial methods with the objective of achieving efficient anti-infection, reducing adverse reactions and, ultimately, achieving good osteogenic effects under infectious conditions.

Huang et al. [41] prepared a Ti_3_C_2_ and CaO_2_ bioheterojunction (MC bio-HJs) by activating cell-like antibacterial strategies, which effectively enhanced the permeability of bacterial membranes. The application of NIR laser irradiation facilitates the penetration of heat and ROS generated by PTT and photodynamic therapy (PDT) through bacterial membranes, leading to substantial disruption of the structural and functional integrity of drug-resistant bacteria and exerting bactericidal effects. The findings demonstrated that the antibacterial efficacy against drug-resistant MRSA and common pathogenic bacteria (*Staphylococcus aureus* and *Escherichia coli*) reached an approximate 100%. In addition to promoting bone integration in vitro, the coating of orthopedic implants was also confirmed to significantly promote bone integration in infected bone defects in two mouse models of in vivo infection.

Deng et al. [42] developed a heterostructure coating comprising Zeolitic imidazolate framework-8 loaded with simvastatin and a PDA nanolayer on a porous bioinert polyetheretherketone implant. The combination of PTT and PDT and the photoinduced acceleration of Zn^2+^ transfer demonstrated effective elimination of both Gram-positive and Gram-negative bacteria. Additionally, superior osteogenic and osseointegration properties were confirmed in vivo.

Red phosphorus (RP) is a material with amorphous structure, IR780 functions as a photosensitizer to generate ROS, and arginine-glycine-asparticacid-cysteine (RGDC) is a peptide which works for promoting osteogenesis. Huang et al. [40] prepared a composite Ti-RP-IR780-RGCC by coating the three materials on the surface of Ti. Irradiation of the composite material with NIR light at a wavelength of 808 nm resulted in an excellent antibacterial effect against *Staphylococcus aureus*. This was achieved through a synergistic effect of PTT and PDT, which effectively eradicated biofilms within the body. Additionally, the material demonstrated excellent osteogenic properties.

### 3.3. Suitable Photothermal Conditions Promote Osteogenesis

PTT based on LRNs can induce mild local photothermal effects and has the characteristics of minimal damage and high accuracy. In recent years, it has been widely used to assist in promoting bone tissue regeneration [43].

**Figure 5:**
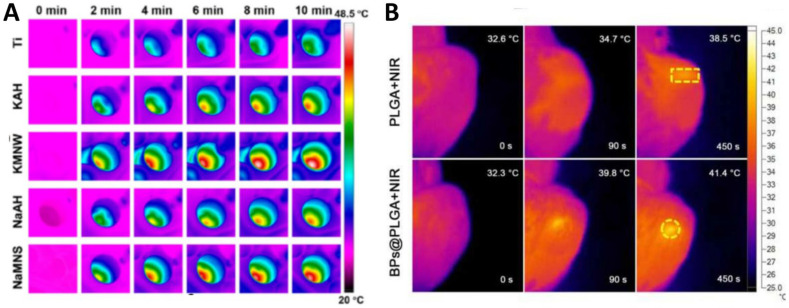
Illustrates the photothermal images. (**A**) Photothermal images of distinct groups subjected to irradiation with an 808 nm laser (0.7 W/cm^2^) [44]. Copyright © 2024, American Chemical Society. (**B**) The infrared thermographic maps of bone defects following implantation indicate the location of the highest temperature in the irradiated areas (dotted box) [45]. Copyright © 2018, Elsevier Ltd.

Wu et al. [44] employed NIR responsive biomimetic micro/nano titanate/TiO_2_-X heterostructure coatings (KMNW and NaMNS) constructed in situ on the surface of Ti implants, achieving local temperatures of 48–51 °C and markedly enhancing the formation of new bone and improving bone integration in vivo (Figure 5A).

Tong et al. [45] have synthesized a novel orthopedic implant material using BP and Poly(lactic-co-glycolic acid) (PLGA) copolymer (BPs@PLGA). The material exhibits a favorable NIR photothermal response and is capable of effectively upregulating the expression of heat shock proteins under suitable photothermal stimulation at 40–42 °C, which promotes bone tissue repair at the site of bone defects (Figure 5B).

Kajiya et al. [46] demonstrated that LRNs, such as carbon nanotubes, can promote the expression of osteogenic genes, including ALP, Runx2, and Osterix, and upregulate heat shock proteins through photothermal effects (NIR at 42 °C for 10 min per day). This results in the promotion of bone tissue regeneration and the achievement of the effect of “photothermal bone promotion”. This has been corroborated in both normal and ovariectomy-induced mouse models of osteoporosis.

The porous silver palladium alloy nanoparticles, designed by Zhang et al. [47], are employed in PTT for in situ bone regeneration. Upon NIR light irradiation, these nanoparticles generate mild local heat (40–43 °C), which significantly accelerates cell proliferation and bone regeneration. The results of the RNA sequencing indicate that the generation of mild local heat under NIR light irradiation can promote bone regeneration by activating the Wnt signaling pathway.

### 3.4. Optoelectronic Microenvironment Promotes Bone Formation

The photoelectric response characteristics of LRNs are of significant importance with regard to the regeneration of bone tissue. LRNs are capable of absorbing light energy and generating electrons, which enables the remote and spatiotemporal precise regulation of cell signal transduction through the adjustment of local cell potential changes. This approach is designed to achieve the objective of non-invasive promotion of bone tissue regeneration [1].

**Figure 6:**
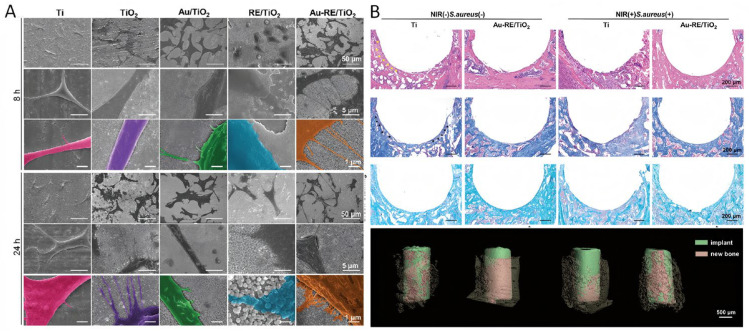
Study on adhesion, vitality, and proliferation of MC3T3-E1 cell surface and osteogenic function in mouse femoral implantation model. (**A**) Cell adhesion, viability, and proliferation of each group surface in vitro with MC3T3-E1 cells. (**B**) 3D reconstruction images of the implants and newly formed bone around the implants. HE staining, Masson staining, and Safranin O-Fast Green staining of the newly formed bone around implants [48]. Copyright © 2023 Wiley-VCH GmbH.

In a study conducted by Tang et al. [48], a photoelectric-responsive Ti implant was designed by loading rare earth elements and gold nanoparticles onto a Ti dioxide nanotube array. Irradiation with NIR resulted in the conversion of energy into hot electrons and lattice vibrations, enhancing the efficiency of the photoelectric conversion process through the energy upconversion effect. The findings of the research demonstrated that the photoresponsive current generated by the photoelectric conversion material system was capable of promoting the proliferation and osteogenic differentiation of MC3T3-E1 cells in vitro, as well as facilitating osseointegration of the implant in vivo (Figure 6).

The ternary nano coating of rGO/g-C_3_N_4_/TiO_2_, prepared by Yan et al. [49] on Ti-based implants, exhibits enhanced visible light-responsive optoelectronic properties, as well as a higher transient photocurrent density and open circuit potential. The application of blue LED light (460 nm) resulted in an enhanced osteogenic differentiation of MC3T3-E1 cells.

In a previous study, Long et al. [50] developed a composite coating comprising GO and TiO_2_ nanodots on the surface of Ti implants. In the presence of visible light, positive charges accumulated on the surface, resulting in a positive surface potential change. This alteration could influence the adhesion behavior of BMSC cells and markedly enhance osteogenic differentiation.

In a study by Fu et al. [51], it was demonstrated that photoresponsive BS/HAP nanofilms are capable of generating photoelectrons following NIR light irradiation. This resulted in an increase in photocurrent and the formation of a rapid and reproducible photoelectronic microenvironment around the implant. The regulation of BMSC behavior can be achieved by modifying the photoelectronic microenvironment. Upon transfer of photoelectrons to the cell membrane of BMSC, a depolarization of the membrane potential occurs, accompanied by a change in cell shape. This, in turn, facilitates calcium influx and activates the Wnt/calcium signaling pathway, which regulates the expression of genes involved in osteogenic differentiation.

### 3.5. Light-Responsive Drug Release System Promotes Bone Function

The field of bone tissue regeneration has seen a growing interest in research on drug release systems based on LRNs, largely due to their capacity to undergo structural alterations under the influence of specific light sources. LRNs are capable of undergoing alterations in their polymerization state or degradation rate when exposed to specific wavelength excitation. This light-controlled release characteristic facilitates the targeted release of drugs at the desired time and location, achieving the objective of targeted therapy. By modulating the intensity, duration, and wavelength of illumination, the release profile and dosage of drugs can be effectively controlled [52]. The release of growth factors or other drugs during the process of bone tissue regeneration and repair can be meticulously adjusted, promoting the proliferation and differentiation of osteoblasts [53].

**Figure 7:**
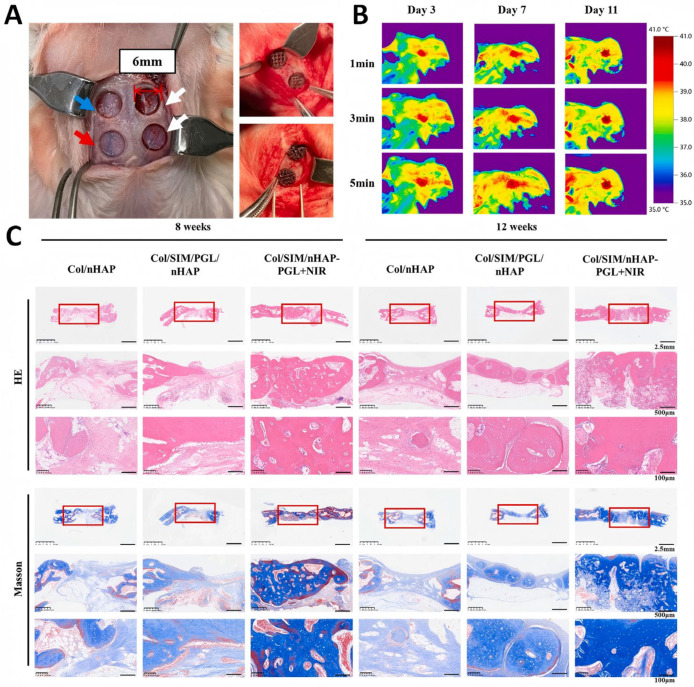
New bone formation capabilities. (**A**) Rabbits underwent cranial defect surgery and were implanted with scaffolds. Blue arrow: Col/nHAP; Red arrow: Col/SIM/PGL/nHAP; White arrow: Col/SIM/nHAP-PGL+NIR. (**B**) Infrared thermal images at cranial defect sites implanted with Col/SIM/nHAP-PGL scaffold and treated with 808 nm NIR light irradiation. (**C**) HE and Masson staining of histological sections at rabbit cranial defect areas at 8 and 12 weeks. The red box is the scaffolds implantation area [54]. Copyright © 2024 Elsevier B.V.

Dong et al. [54] constructed a biomimetic scaffold with dual drug loading of simvastatin and pargyline, achieving the rapid release of simvastatin in the early stages of bone regeneration. During the first 7 days, >65% of simvastatin could be released efficiently, following by a slower and limited drug release within the next 7 days. The controlled release of paraquat can be achieved under NIR irradiation. The experimental results demonstrated that the on-demand release of two drugs effectively accelerated the migration of stem cells, significantly increased ALP activity, significantly upregulated gene expression levels of osteogenic markers, and enhanced the ability of new bone formation in a rabbit skull defect model (Figure 7).

The light-responsive magnesium calcium carbonate microsphere hydrogel composite, prepared by Wan et al. [55], has the capacity to deliver the anti-inflammatory drug aspirin and bone morphogenetic protein-2 (BMP-2) in sequence. The total release of BMP-2 reaches 49.16% at day 14, which maintains an optimal therapeutic concentration of BMP-2 (around 100 ng/mL). In the initial phase, the expeditious release of aspirin mitigates the inflammatory response during the early stages of bone repair, facilitating the transition from the reparative phase to the regenerative phase. In the subsequent phase, the controlled release of BMP-2 is achieved through NIR irradiation, which upregulates the expression of osteogenic pathways to promote bone tissue regeneration in the late stage of bone repair. The results of the Sprague-Dawley rat skull defect model demonstrated a notable enhancement in new bone formation.

The photoresponsive poly(N-isopropylacrylamide benzyl co-nitromethacrylate) (pNIPAm co-NBMA) microgel synthesized by Zhang et al. [56] was loaded with dexamethasone to induce the osteogenic differentiation of hMSCs under UV irradiation and promote bone tissue repair. The release of dexamethasone reached 1.5 ng/μL at 5 min.

As demonstrated by Gu et al. [57], Ti_3_C_2_Tx@PLGA/ICT microspheres can regulate the release of icariin (ICT) through NIR irradiation, promoting the proliferation, osteogenic differentiation, and biomineralization of BMSCs in vitro and osteogenesis and bone integration in rats with bone defects in vivo. The total release of icariin reaches 35.77% at day 14.

### 3.6. Anti-Tumor Effect and Promotion of Osteogenesis

The potential applications of LRNs in anti-tumor therapy and the promotion of bone tissue regeneration are extensive. These materials have the capacity to trigger precise drug release under specific lighting conditions, which facilitates targeted therapy and enhances treatment effectiveness. In the context of anti-tumor therapy, NIR light has the potential to stimulate PTT, enabling nanoparticles to effectively eliminate tumor cells while minimizing damage to surrounding healthy tissues when the local temperature rises [58]. Furthermore, LRNs facilitate the proliferation and differentiation of osteoblasts in bone tissue regeneration by releasing drugs, which accelerates bone healing [22].

Rezk et al. [59] developed a composite nanofiber scaffold consisting of poly (ε-caprolactone) (PCL) and HAP loaded with doxorubicin (DOX) and PDA coatings PCLDH@PDA, which demonstrated a favorable photothermal response under NIR irradiation. The controllable release of DOX was observed to significantly inhibit tumor cell proliferation while also improving the adhesion and proliferation of hMSCs. The results of ALP activity, ARS staining, and biomineralization indicate a promoting effect on calcium deposition and bone cell regeneration.

**Figure 8:**
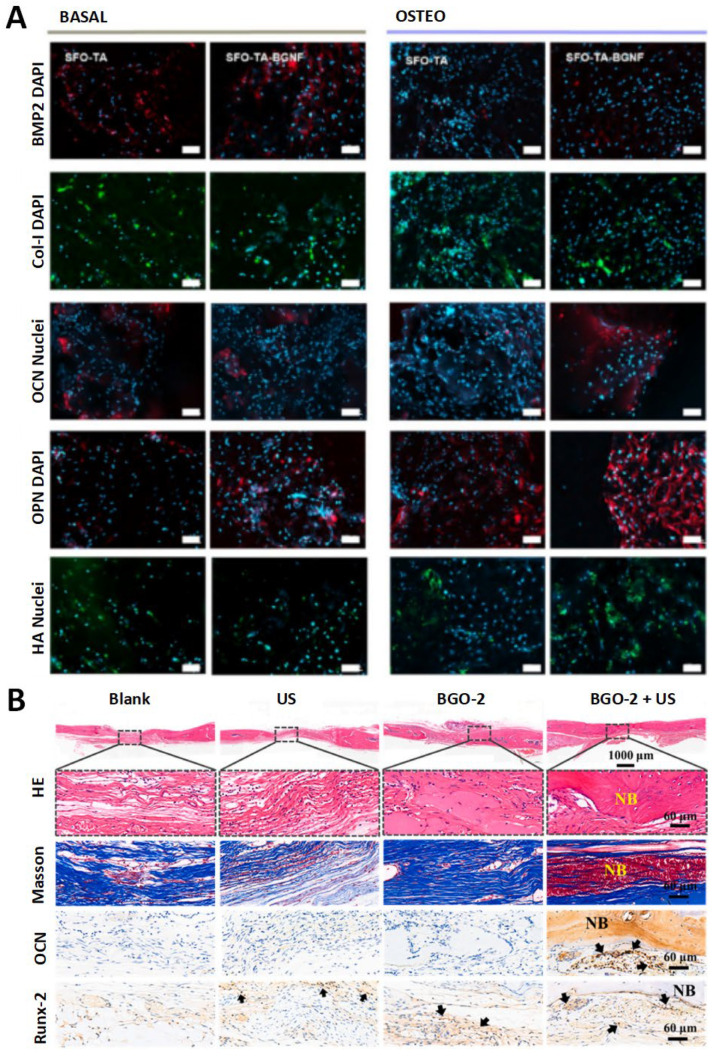
Fluorescence images and histological analysis. (**A**) Bone morphogenic protein-2 (BMP-2, red), collagen type-I α (Col-1α, red), osteopontin (OPN, red), osteocalcin (OCN, red), and hydroxyapatite nanocrystal deposition (HAP, green) after culturing the BM-hMSCs for 21 days. Nuclei counterstained with DAPI (blue). Scale bar: 100 μm [58]. Copyright © 2024, American Chemical Society. (**B**) Histological analysis of bone regeneration in calvarial defects by H&E, MT and immunohistochemical staining of Runx-2 and OCN at 8 weeks after surgery. NB: newly formed bone. The black arrow represents positive staining. Scale bar: 60 μm [60]. Reproduced content is open access. Copyright © 2024 The Authors. Publishing services by Elsevier B.V. on behalf of KeAi Communications Co., Ltd.

The composite aerogel BGNF-PDA, developed by Abie et al. [58], demonstrated a robust photothermal effect and anti-osteosarcoma properties when subjected to NIR laser irradiation. In vitro studies demonstrated that the materials exhibited excellent biocompatibility and osteogenic properties. Within 21 days, the researchers successfully induced stem cells to differentiate into osteoblast-like cells, promoting bone regeneration (Figure 8A).

Xiao et al. [60] have synthesized Bi/SrTiO_3_ nanoheterostructures that are responsive to acidic tumor microenvironments (pH < 6.7), eliminating osteosarcoma cells. The tumor inhibition rates of piezoelectric-enhanced NIR PDT on tibial osteosarcoma models and patient-derived xenograft tumor models were 67.6% and 98.6%, respectively. The PI3K/AKT signaling pathway was activated, resulting in enhanced stability and transcription activity of Runx-2. Additionally, the expression of osteogenic marker genes, including ALP, OPN, and OCN, was upregulated, and osteogenic differentiation of BMSC cells was promoted. The promotion of bone synthesis and metabolism, as well as the facilitation of bone regeneration, was observed in the rat skull defect model (Figure 8B).

The nanohybrid AuNR@CA synthesized by Yang et al. [61] has been demonstrated to trigger apoptosis and inhibit osteosarcoma growth through intense hyperthermia under controlled NIR irradiation. Furthermore, this process has been shown to promote the expression of heat shock proteins and induce significant osteogenic differentiation.

### 3.7. Promote Bone Formation Through Immune Regulation

Macrophages play a pivotal role in the process of bone healing, and maintaining equilibrium in their polarization state is crucial for bone tissue regeneration. It has been demonstrated that the excessive activation of M1 macrophages and the inhibition of M2 macrophage polarization can result in an imbalance of M1/M2 macrophages, which has a significant impact on osteogenesis and angiogenesis in the bone microenvironment. This effect not only delays the bone healing process but may also result in the failure of bone tissue regeneration [62]. In the initial phase of an inflammatory response, macrophages facilitate the transition from the inflammatory stage to the proliferation and remodeling stage by polarizing to the anti-inflammatory phenotype (M2), which is conducive to the regeneration of functional tissues [63]. It is therefore of great clinical significance to regulate the polarization state of M1 and M2 macrophages in a reasonable manner, with the objective of optimizing bone healing through immune regulation.

The biologically active PDA/Ti_3_C_2_/P (VDF-TrFE) nanocomposite coating, developed by Xia et al. [64] with an NIR-triggered photothermal effect, induces macrophage polarization towards the M2 phenotype, reducing the inflammatory response of injured bone tissue, promoting bone regeneration, and creating a favorable microenvironment. This also promotes cell diffusion and growth of BMSCs and significantly upregulates osteogenic markers, including Runx2, ALP, OPN, and OCN.

**Figure 9:**
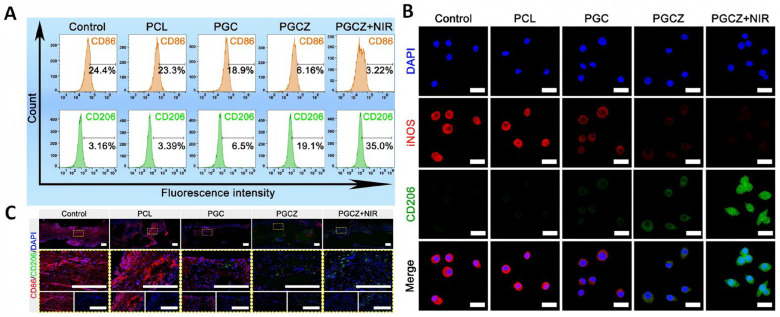
In vitro and in vivo immunomodulatory performance. (**A**) Flow cytometry analysis of macrophage phenotypes after co-culturing for 3 days. (**B**) Immunofluorescence staining images of iNOS and CD206 (red: iNOS; green: CD206; blue: DAPI). Scale bar: 20 μm. (**C**) Immunofluorescence staining images of CD86 and CD206 (red: CD86; green: CD206; blue: DAPI) after 2 weeks of implantation. Scale bar: 200 μm. [65]. Reproduced content is open access. Copyright © 2024 The Authors. Published by Ivyspring International Publisher.

The photoactive soft hard combination scaffold system, as devised by Wu et al. [65], is capable of releasing Zn^2+^ ions in a coordinated manner and promoting macrophage infiltration under periodic NIR irradiation. This effectively regulates the local immune microenvironment and promotes tissue regeneration. The mild hyperthermia induced by NIR irradiation acts in conjunction with the release of Zn^2+^ to reverse high oxidative stress, inhibit M1 macrophage polarization, activate the PI3K/AKT signaling pathway to promote M2 macrophage polarization and related cytokine secretion, promote M1/M2 balance, reduce the inflammatory response, promote angiogenesis and osteogenic differentiation, and accelerate bone formation (Figure 9).

Wu et al. [66] developed an intelligent response photothermal gel platform (GA/BPPD), which is composed of deferoxamine (DFO)-loaded BP nanosheets decorated by a PDA layer (BPPD) and a gelatin methacrylate/sodium alginate methacrylate (GA) hybrid hydrogel. The utilization of NIR photothermal stimulation and pH sensitivity enabled the on-demand release of DFO and PO_4_^3−^, creating a microenvironment conducive to bone regeneration. Under NIR irradiation, the platform effectively promotes osteogenesis and angiogenesis, eliminates ROS, and induces macrophage polarization to the M2 phenotype, improving the immune microenvironment, enhancing functional cytokine secretion, and further promoting tissue regeneration. In vivo experiments have validated the potential of the GA/BPPD system in reducing local inflammation, promoting endogenous cell recruitment, and accelerating revascularization.

In a study by Long et al. [67], a scaffold of PLGA composite BP nanosheets (PLGA/BP scaffold) was prepared using low-temperature 3D printing technology. This scaffold has the ability to recruit macrophages and inhibit inflammation, stimulating M2 polarization of macrophages and promoting bone regeneration. The promotion of osteoblast protein secretion, osteogenic differentiation, and osteogenic behavior is achieved through the PI3K-AKT signaling pathway. The steroid-associated osteonecrosis rat model was employed to investigate its immune regulatory function, which effectively promotes the phenotype of M2 macrophages in vivo, reduces the phenotype of M1 macrophages, and has a positive effect on the formation of new bone in the local anti-inflammatory microenvironment. It has been demonstrated to possess excellent bone regeneration abilities.

## 4. Summary and Discussion

This article provides an overview of the characteristics of LRNs and their potential applications in bone tissue regeneration. The use of LRNs has been demonstrated to facilitate bone tissue repair and regeneration through a number of mechanisms, including their nano-surface morphology, antibacterial properties, osteogenic microenvironment, suitable photothermal effect, photoelectric microenvironment, controllable drug release, anti-tumor effects, and immune regulation. For representative applications of LRNs in bone tissue regeneration, see Table 1.

The potential applications of LRNs in bone tissue regeneration are numerous, yet numerous challenges remain to be overcome in order to facilitate their practical implementation. 1. The issue of biocompatibility and biodegradability is of paramount importance. The primary consideration in the biomedical applications of LRNs is their biocompatibility. It is essential that the material coexists harmoniously with the cells and tissues of the organism, avoiding the triggering of immune reactions or toxicity [68]. Furthermore, the biodegradability of materials is of equal importance. The optimal nanomaterial should be capable of gradual degradation following tissue regeneration, thus avoiding potential adverse effects associated with prolonged retention within the body [69]. It is therefore crucial to select appropriate material matrices and to improve their biocompatibility and biodegradability through modification in order to facilitate their clinical applications. 2. The intensity of the light and its ability to penetrate the depth of the material are also important factors to consider [70,71]. The efficacy of LRNs is contingent upon the intensity and depth penetration capacity of the light source. In practical applications, the absorption and scattering of light by internal tissues can affect the transmission of light, necessitating the development of nanomaterials that can be effectively activated under low-light conditions. Concurrently, to guarantee that the depth of light penetration is adequate to reach the intended tissue, it is essential to investigate light sources with varying wavelengths to enhance the light response characteristics of the material and augment the therapeutic efficacy. 3. The preparation of materials, their stability, and the functionalization of their surfaces are of great importance. The preparation process of nanomaterials has a direct impact on the properties of the resulting material, including particle size, shape, and dispersibility. Furthermore, stability is a pivotal consideration. It is imperative to ensure the stability of materials within the in vivo environment to prevent degradation or deactivation during utilization. Functionalization represents an effective method of enhancing the biological activity of materials. In order to enhance the efficiency with which nanomaterials are applied in the regeneration of bone tissue, researchers must utilize surface modification and other techniques to endow the materials with specific biological functions [72]. 4. The impact of individual differences and the necessity for tailored treatment plans: It is important to consider the potential impact of individual differences, including the physiological and pathological status of each patient, on the efficacy of LRNs. It is important to note that patients may exhibit varying degrees of bone density, regenerative capacity, and light sensitivity. Consequently, the development of personalized treatment plans is essential, with strategies tailored to specific circumstances. This necessitates the undertaking of large-scale population studies during clinical trials, with a view to obtaining data regarding the responses of different populations to light-responsive materials and the optimization of treatment strategies. 5. The efficiency with which light energy is converted: The effective conversion of light energy represents a fundamental aspect of the functionalization of LRNs [73]. The conversion efficiency of materials into biological effects subsequent to the absorption of light energy, such as the promotion of cell proliferation, differentiation, or the induction of bone mineralization, is contingent upon their conversion efficiency. It is evident that there is still scope for enhancement in the conversion efficiency of numerous materials. Consequently, it is imperative to investigate the potential of novel materials or composite materials to optimize the utilization of light energy and augment the efficacy of bone tissue regeneration.

In conclusion, LRNs have demonstrated considerable promise for use in bone tissue regeneration. However, further research and investigation are necessary to address the aforementioned challenges and facilitate their clinical applications.

## Figures and Tables

**Table 1:** Representative applications of LRNs in bone tissue regeneration.

LRN	Application	Reference
Composite material with AgNPs as the core	It promotes cell diffusion and enhances the osteogenic ability and matrix deposition of BMSCs	Chen et al. [26]
TiO_2_ nanotube	It promotes the extension of cytoskeleton and promotes the osteogenic differentiation of hMSCs	Lou et al. [29]
SrTiO_3_ nanorods were coated with a thin layer of NiS to form NiS@SrTiO_3_	It promoted cell adhesion and stimulated MC3T3-E1 cell proliferation and differentiation	Liu et al. [30]
Multifunctional implant coating or integrated bone scaffold with black phosphorus as the core	Photothermal antibacterial, biomineralization and ion release to enhance bone tissue regeneration	Chen et al. [36] Wu et al. [37]
Hydrogenated TiO_2_ nanotube/Ti foil composite	Photodynamic antibacterial, accelerated the adhesion of initial proteins, and promoted the proliferation of MC3T3-E1 cells	Zhao et al. [39]
Composite material with RP as the core	Photothermal and photodynamic synergistically antibacterial, with excellent osteogenic properties	Huang et al. [40]
A novel orthopedic implant material composed of BP and PLGA	Mild photoheat can effectively up-regulate the expression of heat shock proteins and finally promote osteogenesis in vitro and in vivo	Tong et al. [45]
Porous silver palladium alloy nanoparticles	Mild local heat can activate Wnt signaling pathway and promote bone regeneration	Zhang et al. [47]
TiO_2_ nanotube arrays were loaded with rare earth elements and gold nanoparticles	Photoelectrons can remove biofilms and regulate the osteogenesis process	Tang et al. [48]
Go/TiO_2_/Ti thin films	Light-induced surface potential showed a good effect on promoting osteogenic differentiation of bone marrow mesenchymal stem cells and also promoted the adsorption of osteogenic growth factors	Long et al. [50]
BS/HAP nanofilms	Photoelectron promotes calcium influx, activates Wnt/calcium signaling pathway, and regulates downstream genes of osteogenic differentiation	Fu et al. [51]
Calcium carbonate microsphere hydrogel composite	Sequential and controlled release of the anti-inflammatory drugs aspirin and BMP-2 promotes new bone formation	Wan et al. [55]
